# Neurosarcoidosis presenting as longitudinally extensive myelitis: Diagnostic assessment, differential diagnosis, and therapeutic approach

**DOI:** 10.1515/tnsci-2022-0231

**Published:** 2022-07-26

**Authors:** Alessandra Cicia, Viviana Nociti, Assunta Bianco, Chiara De Fino, Vincenzo Carlomagno, Massimiliano Mirabella, Matteo Lucchini

**Affiliations:** Fondazione Policlinico Universitario Agostino Gemellli IRCCS, UOC Neurologia, Rome, Italy; Università Cattolica del Sacro Cuore, Istituto di Neurologia, Centro di Ricerca per la Sclerosi Multipla (CERSM), Largo Agostino Gemelli 8, 00168, Rome, Italy

**Keywords:** neurosarcoidosis, longitudinally extensive transverse myelitis, trident sign, sarcoidosis, treatment

## Abstract

Neurosarcoidosis is an uncommon and multiform clinical entity. Its presentation as an isolated longitudinal extensive transverse myelitis (LETM) is rare and challenging to identify. We report a case of LETM in a 60-year-old patient with no significant systemic symptoms nor relevant medical history. The peculiar spinal magnetic resonance imaging finding characterized by a posterior and central canal subpial contrast enhancement, the so-called “trident sign,” together with chest computed tomography scan and lymph node biopsy led to the diagnosis of sarcoidosis. We also discuss the main differential diagnoses of LETM and therapeutic options for sarcoidosis-related myelitis.

## 1 Introduction

Longitudinal extensive transverse myelitis (LETM) is defined as a spinal cord lesion extending seamlessly over three or more vertebral segments on spinal magnetic resonance imaging (MRI) [1]. LETM is a characteristic feature of neuromyelitis optica (NMO), but can also occur in other conditions affecting the central nervous system (CNS) [2]. Dysimmune demyelinating disorders like acute disseminated encephalomyelitis (ADEM), autoimmune glial fibrillary acidic protein astrocytopathy, and multiple sclerosis can occur as long-segment myelitis; non-inflammatory insults leading to extensive myelopathies include spinal cord compression, artero-venous malformations, and spinal infarction, and need to be differentiated from inflammatory myelitis. Systemic diseases with secondary involvement of the CNS need to be considered as well. Among them, rheumatologic disorders such as systemic lupus erythematosus (SLE), Behcet’s disease, and sarcoidosis can involve both brain and spinal cord. Occasionally, neurologic involvement may represent the only sign of those diseases, whose identification and accurate diagnosis remains a challenging issue.

We report the case of a patient presenting with a longitudinally extensive myelitis as the exclusive symptom of sarcoidosis, and discuss the spectrum of differential diagnosis of LETM, the main MRI features and clinical clues that can orientate the diagnostic process, and provide an overview of the current treatment options.

## 2 Case report

A 60-year-old man with no relevant past medical history was admitted to our department for the appearance of pain in the right hemi-abdomen, followed by paresthesias in the lower limbs and in the trunk. The neurological examination revealed pyramidal signs, with brisk deep tendon reflexes at the lower limbs, ankle clonus, and bilateral Babinski sign.

**Figure 1:**
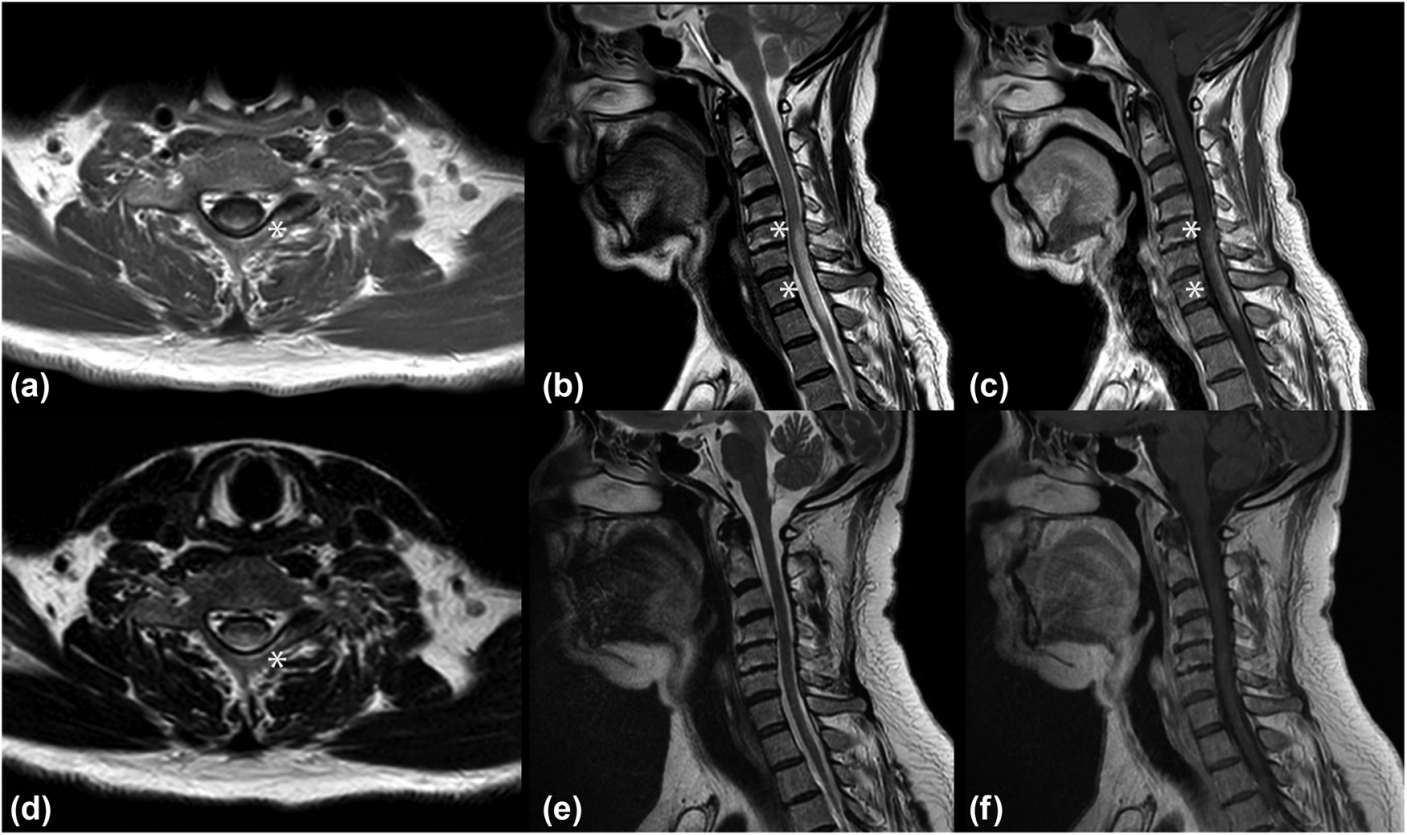
(a–d) Spinal MRI at clinical onset. (b) Sagittal T2-weighted image showing the hyperintense lesion extending from C5 to D2 (asterisks); (c) Sagittal T1 post-contrast sequence showing posterior subpial contrast enhancement (asterisks). Axial T1 post-contrast (a) and t2-weighted (d) sequences at symptoms’ onset showing the “trident sign” (asterisk). (e and f) spinal MRI 6 months after treatment. Sagittal T2-weighted (e) and T1 post-contrast (f) sequences showing an almost complete disappearance of the spinal lesion.

Cervical and dorsal spine MRI showed a hyperintensity area extended from C5 to D2 on T2-weighted images, involving both the gray and white matter. Post-contrast scans revealed inhomogeneous enhancement of the lesion, distributed both in the dorsal subpial space and inside the central canal (Figure 1). Brain MRI resulted negative.

**Figure 2:**
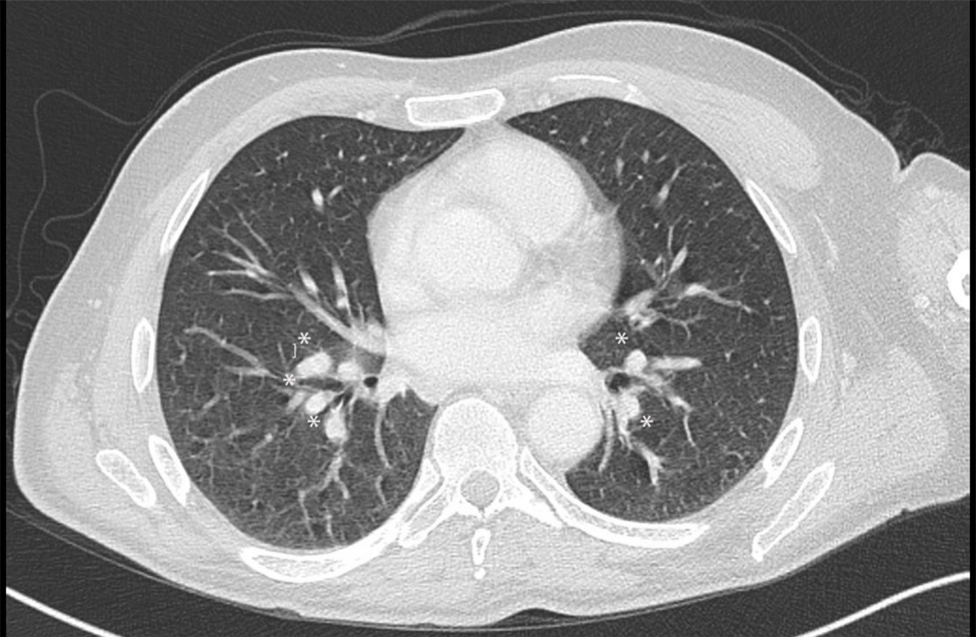
Axial chest CT scan showing bilateral hilar adenopathy (asterisks).

Cerebrospinal fluid (CSF) analysis showed mildly elevated protein level (48 mg/dL) with normal glycorrhachia and no cells. Oligoclonal bands were absent. Anti-AQP4 and anti-MOG antibodies in serum, tested through a cell-based assay (CBC), were not detected. To exclude paraneoplastic etiology, a total body computed tomography (CT) was performed. Despite the complete absence of respiratory symptoms, the thoracic CT scan showed multiple enlarged lymph nodes with central colliquation in mediastinal and hilar stations (Figure 2). The histological analysis conducted on the transbronchial needle biopsy demonstrated granulomatous lymphadenitis with non-caseating granulomas supportive for the diagnosis of sarcoidosis. Based on the criteria proposed by Zajicek et al., our patient fitted the criteria of probable neurosarcoidosis (NS) [3].

The patient was treated with high doses of intravenous corticosteroid therapy for 5 days (methylprednisolone 1 g per day) and after discharge with tapering doses of oral corticosteroids starting from prednisone 50 mg per day with slow reduction until suspension after 9 months. A new MRI of the cervical and dorsal spine was performed 6 weeks later showing the almost complete disappearance of the inflammatory lesion and the absence of contrast enhancement (Figure 1). Mycophenolate mofetil was added 3 months later. A thoracic CT scan performed 1 year later showed the reduction in the mediastinal and hilar lymph nodes. No clinical or radiological relapses were reported in the two following years.


**Ethical approval:** The research related to human use has been complied with all the relevant national regulations, institutional policies and in accordance with the tenets of the Helsinki Declaration, and has been approved by the authors’ institutional review board or equivalent committee.
**Informed consent:** Informed consent has been obtained from all individuals included in this study.

## 3 Discussion

Sarcoidosis is a multisystem granulomatous disorder characterized by the presence of non-caseating granulomas in the involved organs. It is diffused worldwide, and its prevalence varies from 1–5 per 100,000 in the Far East to 140–160 per 100,000 in Sweden and Canada [4].

The involvement of CNS establishes a picture of NS, which occurs in approximately 5–16% of patients affected by sarcoidosis [5]. NS can arise with a wide variety of clinical presentations, most commonly cranial and peripheral neuropathy, aseptic meningitis, and psychiatric symptoms. The diagnosis of NS is established by the clinical syndrome, imaging and histopathological findings, and exclusion of other causes. There are no specific laboratory findings and CSF analysis shows in most cases an increase in cell count and protein levels (58–63% of cases of NS in one meta-analysis), while hypoglycorrhachia is infrequently seen (14% of cases) [6].

Moreover, CSF abnormalities can be more pronounced in patients with diffuse leptomeningeal enhancement on MRI [7].

Neurologic symptoms, including LETM, may be the presenting feature of sarcoidosis in one-half of individuals with sarcoidosis [8]. Approximately 75% of patients with systemic sarcoidosis who develop NS will do so within 2 years of sarcoidosis diagnosis [9].

NS presentation as LETM is a rare occurrence, with an incidence estimated at 0.43–1% of all patients with sarcoidosis [10,11]. It represents an important diagnostic challenge since it mimics other inflammatory or non-inflammatory neurologic diseases, such as NMO, multiple sclerosis (MS), spinal cord tumors, and arteriovenous fistulas, and sometimes occurs in the absence of other evocative systemic symptoms of sarcoidosis.

LETM refers to a subtype of acute transverse myelitis appearing at the MRI as a continuous lesion of at least three vertebral segments in length. When coping with longitudinally extensive myelitis, a detailed history and a thorough physical examination are indispensable (Table 1). The location and length of the lesion on MRI are important discriminators with etiologic and prognostic significance.

**Table 1:** Differential diagnosis of LETM

Disease	LETM frequency	MRI features	Other diagnostic clues
NMOSD	62–85% [17]	Central lesions, frequently located in the thoracic cord [14]. Bright spotty lesions in T2 axial scans and lens-shaped pattern of enhancement on sagittal images	History of recurrent optic neuritis or myelitis, VEP, AQP4/MOG on serum
MS	Unusual [40]	Peripheral lesions frequently located in the cervical cord [18]. Patchy, nodular, or ring-shaped enhancement in acute phases, usually resolving within 2 months. Confluent short lesions may be misinterpreted as LETM	Ovoidal white matter lesions in suggestive areas [41]; OCB in CSF; clinical or radiological evidence of dissemination in space and time
ADEM	up to 33% [42]	Gray-white matter involvement with tumefactive appearance	More prevalent in children; temporal relation with a vaccination or infection; evidence of encephalopathy; leukocytosis on CSF
Paraneoplastic myelitis	Rare	T2 hyperintensities of the dorsal and lateral columns in axial scans; symmetrical enhancement in post-contrast scans (“owl eye” sign) [23].	Systemic symptoms or signs of malignancy; body CT scan; serum and CSF onconeural antibody panel. SCLC, thymoma, breast, ovary, and endometrium cancer more often involved.
Compressive myelopathy	Rare	Lesions often involve cervical cord, and show a pancake-like focal enhancement; spine lesions are associated with degenerative disk disease	History of trauma or osteo-disk arthrosis; gradual onset of symptoms; scarce response to corticosteroids
DAVF	Rare	T2-hyperintense lesions with diffuse edema more often located in the thoracolumbar cord [43]; involvement of the conus medullaris in up to 90% of cases because of orthostasis; presence of flow voids; patchy intramedullary enhancement and serpentine enhancing veins on the cord surface	Usually elderly men; symptoms worsen with erect posture or Valsalva maneuvre
Spinal cord infarction	Rare cause of stroke [44]	*Anterior spinal artery (Adamkiewicz artery)*: on axial scans snake-eyes sign; symmetric circular or ovoid foci of T2 hyperintensity in the anterior horns of the spinal cord; on sagittal scans pencil-like sign: vertical linear high T2 hyperintensity. *Posterior spinal arteries*: usually unilateral lesions confined to the dorsal columns [45]. DWI restriction may be limited by CSF flow artefact	Acute onset risk factors for atherosclerosis, cardioembolism, history of hypertension, aortic surgery, or acute disc herniation
Neuro-Behçet’s	10–18% [46]	T2 hyperintense lesions at the cervico-medullary or ponto-medullary junctions, associated with vasogenic edema; moderate patchy enhancement	Recurrent oral aphthae, genital ulcers, arthritis, uveitis, thrombophlebitis; pathergy test; chest and joint radiographs; detection of HLA-B51
NS	0.43–1% [27]	Smooth or nodular leptomeningeal enhancement with patchy peripheral cord enhancement; trident sign: combination of linear dorsal subpial enhancement associated with central canal contrast uptake in axial sequences. Persistence of enhancement beyond 3 months (vs MS)	Chest RX or CT: enlarged lymph nodes within mediastinal and hilar stations; increased serum and CSF ACE level (not pathognomonic) Systemic involvement: erythema nodosum, arrhythmias, uveitis, IBD-like disorders, blood dyscrasia

LETM: longitudinally extensive transverse myelitis; NMOSD: neuromyelitis optica spectrum disorders; VEP: visual evoked potentials; AQP4: aquaporin 4; MOG: myelin oligodendrocyte glycoprotein; OCB: oligoclonal bands; CSF: cerebrospinal fluid; ADEM: acute demyelinating encephalomyelitis; SCLC: small cells lung cancer; DWI: diffusion weighted imaging; ACE: angiotensin converting enzyme; MS: multiple sclerosis; IBD: inflammatory bowel disease; MRI: magnetic resonance imaging; DAVF: dural arteriovenous fistulas; NS: neurosarcoidosis; RX: radiography; CT: computerized tomography.

LETM is typically associated with neuromyelitis optica spectrum disorders (NMOSD), as it represents the inaugural form of the disease in a high proportion of patients and also it is one of its diagnostic criteria [12]. In a high percentage of patients affected by NMOSD, antibodies against aquaporin-4 (anti-AQP4) or, less frequently, for myelin oligodendrocyte glycoprotein (anti-MOG) can be detected in the serum [13]. The typical imaging findings of the NMOSD-associated LETM include central lesions, frequently located in the thoracic cord [14], enhancing in post-contrast images with a patchy, ring-like, or open ring pattern. “Bright spotty lesions” in axial T2-weighted images have been described as relatively specific for distinguishing NMO from other entities including MS [15,16]. In MOG-IgG-associated myelitis (MOGAD), the involvement of the conus medullaris is more frequent than in anti-AQP4-associated NMO; moreover, in MOGAD, contrast enhancement of the lesions is less common, but if present, it is very characteristic to find a distinctive H pattern [17].

Despite the strong association between LETM and NMOSD, a variety of other different conditions may present with LETM [1]. MS is the most common cause of short segment myelitis and attention must be paid to the cases of coalescent multiple short T2 lesions, which may be misinterpreted as LETM on sagittal images. In those cases, axial images are useful to distinguish the adjacent, discrete lesions. When occurring as LETM, MS lesions are usually located in the cervical cord with a peripheral, mostly dorso-lateral distribution and this finding favors MS rather than NMOSD [18,19]. Extensive myelitis can also occur as part of an ADEM, often following an infection or vaccination. Recently, cases of LETM associated with SARS-CoV-2 infection have been described, both as para-infectious or post-infectious acute myelitis [20]. In addition, a few cases of LETM following anti-COVID vaccination have been described [21,22]. Paraneoplastic syndromes can also present as extensive myelitis that often precedes the diagnosis of cancer. In those cases, a typical MRI feature is the so-called “owl eye,” which refers to the symmetrical enhancement of the dorsal and lateral columns, evident in the axial view. Anti-amphiphysin and anti-collapsin response mediator protein 5 immunoglobulin G (IgG) are the most frequent neural autoantibodies associated with paraneoplastic myelopathies being most commonly associated with small cell lung cancer and breast cancer. Paraneoplastic myelitis associated with anti-glutamic acid decarboxylase, ANNA-1, and anti-Yo antibodies have also been described [23].

As for non-inflammatory causes, subacute or chronic compressive myelopathy, often secondary to cervical spondylosis [24], is a rather common cause of focal myelopathy. More rarely, a vast stenosis involving multiple vertebral segments may resemble an extensive myelitis on MRI. A “pancake-like” focal enhancement, due to the disruption of the blood–brain barrier at the point of maximal stenosis, may be evocative of compressive myelitis.

Moreover, vascular abnormalities like dural arteriovenous fistulas (DAVF), typically located in the thoracolumbar region, can mimic an inflammatory myelopathy. They can be revealed by the detection of “flow voids” on the surface of the caudal spinal cord in T2 weighted MRI, reflecting dilated veins [25]. Infectious causes of extensive myelitis include borreliosis and, more rarely, tuberculosis and cryptococcosis. In the differential diagnosis, neoplastic causes like CNS lymphomas should also be considered.

Systemic autoimmune diseases, such as SLE and Sjӧgren syndrome can affect the CNS and cause myelitis. Behcet’s disease is rarely associated with longitudinal cord lesions and more often they are located at the cervical-medullary or pontomedullary junctions [26].

Alike sarcoidosis, the imaging spectrum for spinal sarcoidosis is wide, but most commonly includes smooth or nodular leptomeningeal enhancement with patchy peripheral cord enhancement, due to inflammatory infiltration of the perivascular spaces. Isolated involvement of the spinal cord is rare (<0.5% of sarcoidosis cases) and linear dorsal subpial enhancement can be present at the MRI [27,28]. A combination of linear dorsal subpial and central canal enhancement in axial sequences depicts the so-called trident sign. This peculiar MRI feature is now widely accepted as a clue sign of spinal cord sarcoidosis and may offer valuable support in doubtful cases [29]. In this regard, it is indicative in the case reported by Jolliffe et al., who described a patient affected by LETM initially diagnosed as NMOSD because of a positive ELISA testing for anti-AQP4 IgG, but in whom detection of the trident sign in the spinal MRI finally led to the corrected diagnosis of spinal cord sarcoidosis [30].

When managing extensive myelitis, pharmacological treatment should be guided by the underlying etiology.

Except for some patients with incidental and asymptomatic hilar/mediastinal lymphadenopathy (who still require strict monitoring), sarcoidosis management requires the prompt initiation of pharmacological treatment with steroids eventually associated with immunomodulatory drugs, especially in case of extra-pulmonary involvement [31]. To date, treatment of sarcoidosis is largely guided by small, uncontrolled trials and expert consensus, due to the rarity of the disease and the heterogeneity of clinical presentation [32].

The question of whether the involvement of specific organs may respond to a particular steroid-sparing agent is not wholly clear, and generally extrapulmonary sarcoidosis is treated with the same algorithms as pulmonary sarcoidosis [31]. Corticosteroids are considered first-line treatment for all types of sarcoidosis, at a starting dosage of 20–40 mg of prednisone per day with higher doses in cases of severe NS or severe damage of other organs [33].

Corticosteroid-sparing medications like methotrexate, azathioprine, leflunomide, and mycophenolate may be added in chronic or particularly aggressive forms. Early use of TNF inhibitors like infliximab should be also considered following the report of their significant effectiveness [34]. Mycophenolate mofetil was the therapy chosen in our patient; it has demonstrated to be effective for NS with central involvement and to have a better tolerability profile than other immunosuppressive options in one series [35]. Duration of therapy, whether with corticosteroids alone or in association with other immunosuppressive treatment, is generally recommended approximately for 1 year, based on data suggesting an increased risk of relapse with shorter courses [36]. A dual therapy encompassing both glucocorticoids and other immunosuppressants seems to be particularly important to prevent neurologic relapses [37,38].

Altogether sarcoidosis treatment is complex and non-standardized for clinicians and patients, thus an interprofessional approach is indispensable to tailor the best therapy for each patient, depending on organ involvement, drug toxicity profile, and individual tolerability.

## 4 Conclusion

The low prevalence of spinal cord sarcoidosis, the wide range of possible differential diagnosis and the absence of symptomatic systemic involvement often make it challenging to identify this condition, leading to delay in diagnosis and treatment with an increase in morbidity.

Adjunctive laboratory tests may not be reliable, since many of the possible findings are not specific nor adequately sensitive (e.g., increased protein level or pleocytosis in CSF and ACE enzyme increase in serum).

There are no definitive diagnostic features of NS on imaging, but the experience reported in our patient, in accordance with current literature, demonstrates that in the presence of an isolated transverse myelitis, it is worth extending clinical assessment to other organs, in search of systemic diseases. MRI features, namely, the enhancement pattern presented by a spinal lesion, constitute an important clue to direct the diagnostic process [39]. The trident sign is by now a precious hint to raise suspicion of NS.

## References

[1] Tobin WO, Weinshenker BG, Lucchinetti CF. Longitudinally extensive transverse myelitis. Curr Opin Neurol. 2014;27:279–89. 10.1097/WCO.0000000000000093 24792338

[2] Wingerchuk DM, Banwell B, Bennett JL, Cabre P, Carroll W, Chitnis T, et al. International consensus diagnostic criteria for neuromyelitis optica spectrum disorders. Neurology. 2015;85:177–89. 10.1212/WNL.0000000000001729 PMC4515040 26092914

[3] Zajicek JP, Scolding NJ, Foster O, Rovaris M, Evanson J, Moseley IF, et al. Central nervous system sarcoidosis-diagnosis and management. QJM. 1999;92:103–17. 10.1093/qjmed/92.2.103 10209662

[4] Arkema EV, Cozier YC. Sarcoidosis epidemiology: recent estimates of incidence, prevalence and risk factors. Curr Opin Pulm Med. 2020;26:527–34. 10.1097/MCP.0000000000000715 PMC7755458 32701677

[5] Hoitsma E, Faber CG, Drent M, Sharma OP. Neurosarcoidosis: a clinical dilemma. Lancet Neurol. 2004;3:397–407. 10.1016/S1474-4422(04)00805-1 15207796

[6] Fritz D, van de Beek D, Brouwer MC. Clinical features, treatment and outcome in neurosarcoidosis: systematic review and meta-analysis. BMC Neurol. 2016;16:220. 10.1186/s12883-016-0741-x PMC5109654 27846819

[7] Wengert O, Rothenfusser-Korber E, Vollrath B, Bohner G, Scheibe F, Otto C, et al. Neurosarcoidosis: correlation of cerebrospinal fluid findings with diffuse leptomeningeal gadolinium enhancement on MRI and clinical disease activity. J Neurol Sci. 2013;335:124–30. 10.1016/j.jns.2013.09.008 24071064

[8] Stern BJ, Krumholz A, Johns C, Scott P, Nissim J. Sarcoidosis and its neurological manifestations. Arch Neurol. 1985;42:909–17. 10.1001/archneur.1985.04060080095022 3896208

[9] Krumholz A, Stern BJ. Neurologic manifestations of sarcoidosis. Handb Clin Neurol. 2014;119:305–33. 10.1016/B978-0-7020-4086-3.00021-7 24365304

[10] Duhon BS, Shah L, Schmidt MH. Isolated intramedullary neurosarcoidosis of the thoracic spine: case report and review of the literature. Eur Spine J. 2012;21;(Suppl 4):S390–5. 10.1007/s00586-011-1842-2 PMC3369059 21598117

[11] Wang L, Li Y. Longitudinal ultra-extensive transverse myelitis as a manifestation of neurosarcoidosis. J Neurol Sci. 2015;355:64–7. 10.1016/j.jns.2015.05.017 26027786

[12] Wingerchuk DM, Lennon VA, Lucchinetti CF, Pittock SJ, Weinshenker BG. The spectrum of neuromyelitis optica. Lancet Neurol. 2007;6:805–15. 10.1016/S1474-4422(07)70216-8 17706564

[13] Flanagan EP. Neuromyelitis optica spectrum disorder and other non-multiple sclerosis central nervous system inflammatory diseases. Contin (Minneap Minn). 2019;25:815–44. 10.1212/CON.0000000000000742 31162318

[14] Kim HJ, Paul F, Lana-Peixoto MA, Tenembaum S, Asgari N, Palace J, et al. MRI characteristics of neuromyelitis optica spectrum disorder: an international update. Neurology. 2015;84:1165–73. 10.1212/WNL.0000000000001367 PMC4371410 25695963

[15] Dutra BG, da Rocha AJ, Nunes RH, Maia ACMJ. Neuromyelitis optica spectrum disorders: Spectrum of MR imaging findings and their differential diagnosis. Radiographics. 2018;38:169–93. 10.1148/rg.2018170141 29320331

[16] Yonezu T, Ito S, Mori M, Ogawa Y, Makino T, Uzawa A, et al. “Bright spotty lesions” on spinal magnetic resonance imaging differentiate neuromyelitis optica from multiple sclerosis. Mult Scler. 2014;20:331–7. 10.1177/1352458513495581 23828869

[17] Dubey D, Pittock SJ, Krecke KN, Morris PP, Sechi E, Zalewski NL, et al. Clinical, radiologic, and prognostic features of myelitis associated with myelin oligodendrocyte glycoprotein autoantibody. JAMA Neurol. 2019;76:301–9. 10.1001/jamaneurol.2018.4053 PMC6440233 30575890

[18] Dumrikarnlert C, Siritho S, Chulapimphan P, Ngamsombat C, Satukijchai C, Prayoonwiwat N. The characteristics of spinal imaging in different types of demyelinating diseases. J Neurol Sci. 2017;372:138–43. 10.1016/j.jns.2016.11.035 28017200

[19] Flanagan EP, Lennon VA, Pittock SJ. Autoimmune myelopathies. Contin (Minneap Minn). 2011;17:776–99. 10.1212/01.CON.0000403795.20914.6c 22810931

[20] Mondal R, Deb S, Shome G, Ganguly U, Lahiri D, Benito-León J. COVID-19 and emerging spinal cord complications: A systematic review. Mult Scler Relat Disord. 2021;51:102917. 10.1016/j.msard.2021.102917 PMC7981271 33845350

[21] Notghi AA, Atley J, Silva M. Lessons of the month 1: Longitudinal extensive transverse myelitis following AstraZeneca COVID-19 vaccination. Clin Med (Lond). 2021;21:e535–8. 10.7861/clinmed.2021-0470 PMC8439525 34507942

[22] Pagenkopf C, Südmeyer M. A case of longitudinally extensive transverse myelitis following vaccination against Covid-19. J Neuroimmunol. 2021;358:577606. 10.1016/j.jneuroim.2021.577606 PMC8223023 34182207

[23] Flanagan EP, McKeon A, Lennon VA, Kearns J, Weinshenker BG, Krecke KN, et al. Paraneoplastic isolated myelopathy: clinical course and neuroimaging clues. Neurology. 2011;76:2089–95. 10.1212/WNL.0b013e31821f468f 21670438

[24] Flanagan EP, Marsh RW, Weinshenker BG. Teaching neuroimages: “pancake-like” gadolinium enhancement suggests compressive myelopathy due to spondylosis. Neurology. 2013;80:e229. 10.1212/WNL.0b013e318293e346 23690303

[25] Atkinson JL, Miller GM, Krauss WE, Marsh WR, Piepgras DG, Atkinson PP, et al. Clinical and radiographic features of dural arteriovenous fistula, a treatable cause of myelopathy. Mayo Clin Proc. 2001;76:1120–30. 10.4065/76.11.1120 11702900

[26] Graham D, McCarthy A, Kavanagh E, O’Rourke K, Lynch T. Teaching NeuroImages: longitudinally extensive transverse myelitis in neuro-Behcet disease. Neurology. 2013;80:e189–90. 10.1212/WNL.0b013e3182904d2e 23628935

[27] Flanagan EP, Kaufmann TJ, Krecke KN, Aksamit AJ, Pittock SJ, Keegan BM, et al. Discriminating long myelitis of neuromyelitis optica from sarcoidosis. Ann Neurol. 2016;79:437–47. 10.1002/ana.24582 26677112

[28] Kasliwal MK, Harbhajanka A, Nag S, O’Toole JE. Isolated spinal neurosarcoidosis: An enigmatic intramedullary spinal cord pathology-case report and review of the literature. J Craniovertebr Junction Spine. 2013;4:76–81. 10.4103/0974-8237.128536 PMC3980561 24744567

[29] Zalewski NL, Krecke KN, Weinshenker BG, Aksamit AJ, Conway BL, McKeon A, et al. Central canal enhancement and the trident sign in spinal cord sarcoidosis. Neurology. 2016;87:743–4. 10.1212/WNL.0000000000002992 27527540

[30] Jolliffe EA, Keegan BM, Flanagan EP. Trident sign trumps Aquaporin-4-IgG ELISA in diagnostic value in a case of longitudinally extensive transverse myelitis. Mult Scler Relat Disord. 2018;23:7–8. 10.1016/j.msard.2018.04.012 29709797

[31] Gerke AK. Treatment of sarcoidosis: a multidisciplinary approach. Front Immunol. 2020;11:545413. 10.3389/fimmu.2020.545413 PMC7732561 33329511

[32] Brito-Zerón P, Pérez-Alvarez R, Pallarés L, Retamozo S, Baughman RP, Ramos-Casals M. Sarcoidosis: an update on current pharmacotherapy options and future directions. Expert Opin Pharmacother. 2016;17:2431–48. 10.1080/14656566.2016.1258061 27817209

[33] Grutters JC, van den Bosch JM. Corticosteroid treatment in sarcoidosis. Eur Respir J. 2006;28:627–36. 10.1183/09031936.06.00105805 16946094

[34] Gelfand JM, Bradshaw MJ, Stern BJ, Clifford DB, Wang Y, Cho TA, et al. Infliximab for the treatment of CNS sarcoidosis: A multi-institutional series. Neurology. 2017;89:2092–100. 10.1212/WNL.0000000000004644 PMC5711506 29030454

[35] Androdias G, Maillet D, Marignier R, Pinède L, Confavreux C, Broussolle C, et al. Mycophenolate mofetil may be effective in CNS sarcoidosis but not in sarcoid myopathy. Neurology. 2011;76:1168–72. 10.1212/WNL.0b013e318212aafb 21444902

[36] Baughman RP, Judson MA. Relapses of sarcoidosis: what are they and can we predict who will get them? Eur Respir J. 2014;43:337–9. 10.1183/09031936.00138913 24488991

[37] Russell E, Luk F, Manocha S, Ho T, O’Connor C, Hussain H. Long term follow-up of infliximab efficacy in pulmonary and extra-pulmonary sarcoidosis refractory to conventional therapy. Semin Arthritis Rheum. 2013;43:119–24. 10.1016/j.semarthrit.2012.10.008 23332903

[38] Kidd DP. Neurosarcoidosis: clinical manifestations, investigation and treatment. Pract Neurol. 2020;20:199–212. 10.1136/practneurol-2019-002349 32424017

[39] Mustafa R, Passe TJ, Lopez-Chiriboga AS, Weinshenker BG, Krecke KN, Zalewski NL, et al. Utility of MRI enhancement pattern in myelopathies with longitudinally extensive T2 lesions. Neurol Clin Pract. 2021;11:e601–11. 10.1212/CPJ.0000000000001036 PMC8610516 34824894

[40] Asnafi S, Morris PP, Sechi E, Pittock SJ, Weinshenker BG, Palace J, et al. The frequency of longitudinally extensive transverse myelitis in MS: A population-based study. Mult Scler Relat Disord. 2020;37:101487. 10.1016/j.msard.2019.101487 31707235

[41] Filippi M, Rocca MA, Ciccarelli O, De Stefano N, Evangelou N, Kappos L, et al. MRI criteria for the diagnosis of multiple sclerosis: MAGNIMS consensus guidelines. Lancet Neurol. 2016;15:292–303. 10.1016/S1474-4422(15)00393-2 PMC4760851 26822746

[42] Sarbu N, Shih RY, Jones RV, Horkayne-Szakaly I, Oleaga L, Smirniotopoulos JG. White matter diseases with radiologic-pathologic correlation. Radiographics. 2016;36:1426–47. 10.1148/rg.2016160031 27618323

[43] Gilbertson JR, Miller GM, Goldman MS, Marsh WR. Spinal dural arteriovenous fistulas: MR and myelographic findings. AJNR Am J Neuroradiol. 1995;16:2049–57. PMC8337217 8585493

[44] Nedeltchev K, Loher TJ, Stepper F, Arnold M, Schroth G, Mattle HP, et al. Long-term outcome of acute spinal cord ischemia syndrome. Stroke. 2004;35:560–5. 10.1161/01.STR.0000111598.78198.EC 14726546

[45] Masson C, Pruvo JP, Meder JF, Cordonnier C, Touzé E, De La Sayette V, et al. Spinal cord infarction: clinical and magnetic resonance imaging findings and short term outcome. J Neurol Neurosurg Psychiatry. 2004;75:1431–5. 10.1136/jnnp.2003.031724 PMC1738740 15377691

[46] Brown JWL, Coles A, Horakova D, Havrdova E, Izquierdo G, Prat A, et al. Association of initial disease-modifying therapy with later conversion to secondary progressive multiple sclerosis. Jama. 2019;321:175–87. 10.1001/jama.2018.20588 PMC6439772 30644981

